# *Limosilactobacillus fermentum* JL-3 isolated from “Jiangshui” ameliorates hyperuricemia by degrading uric acid

**DOI:** 10.1080/19490976.2021.1897211

**Published:** 2021-03-25

**Authors:** Ying Wu, Ze Ye, Pengya Feng, Rong Li, Xiao Chen, Xiaozhu Tian, Rong Han, Apurva Kakade, Pu Liu, Xiangkai Li

**Affiliations:** a Gansu Key Laboratory of Biomonitoring and Bioremediation for Environmental Pollution, School of Life Sciences, Lanzhou University, Lanzhou, China; b Ministry of Education Key Laboratory of Cell Activities and Stress Adaptations, School of Life Science, Lanzhou University, Lanzhou, China

**Keywords:** Hyperuricemia, gout, *limosilactobacillus fermentum*, uric acid, gut microbiota

## Abstract

Recent studies into the beneficial effects of fermented foods have shown that this class of foods are effective in managing hyperuricemia and gout. In this study, the uric acid (UA) degradation ability of *Limosilactobacillus fermentum* JL-3 strain, isolated from “Jiangshui” (a fermented Chinese food), was investigated. *In vitro* results showed that JL-3 strain exhibited high degradation capacity and selectivity toward UA. After oral administration to mice for 15 days, JL-3 colonization was continuously detected in the feces of mice. The UA level in urine of mice fed with JL-3 was similar with the control group mice. And the serum UA level of the former was significantly lower (31.3%) than in the control, further confirmed the UA-lowering effect of JL-3 strain. *Limosilactobacillus fermentum* JL-3 strain also restored some of the inflammatory markers and oxidative stress indicators (IL-1β, MDA, CRE, blood urea nitrogen) related to hyperuricemia, while the gut microbial diversity results showed that JL-3 could regulate gut microbiota dysbiosis caused by hyperuricemia. Therefore, the probiotic *Limosilactobacillus fermentum* JL-3 strain is effective in lowering UA levels in mice and could be used as a therapeutic adjunct agent in treating hyperuricemia.

## 1. Introduction

Hyperuricemia is one of the major metabolic diseases associated with gout which is on a rise in many countries.^1–3^ Some studies have reported that the prevalence of gout is from 1% to 3% in Europe and 3.9% in the US.^4,5^ Also, more than 7.44 million cases of gout were estimated to have occurred worldwide in 2017.^6^ According to the Gout Report White Paper (2017), hyperuricemia patients in China had reached 170 million, of which approximately 47% developed gout with an annual growth rate of 9.7%.^7^ Foods rich in the purines are incompletely degraded to UA in humans, resulting in high levels of UA in the plasma.^8,9^ Hence, UA accumulation in tissues as urate, which had been considered as an important risk factor for the development of gout and other diseases such as cardiovascular disease, endothelial dysfunction and metabolic syndrome.^10,11^

High levels of UA in plasma have long been associated with hyperuricemia.^12^ There are two ways of UA accumulation in humans. First is an endogenous buildup caused by enzyme deficiencies in the purine metabolism pathway. This deficiency in major purine metabolism enzymes leads to an increased rate of nucleic acid decomposition and UA production, which is under the control of a mechanism whereby an end product control itself by inhibiting the enzyme producing it.^13^ Another route of UA accumulation is through the intake and exogenous absorption of purine rich foods. A recent study showed that the intake of meat increases the risk of gout by 21%, while sea food intake increases its risk by just 7%.^14^ Hyperuricemia can be alleviated effectively by external interventions.^15^ In general, it can also be managed through the excretion of UA in large amounts by the kidneys, while only 30% of the total UA is degraded by the action of intestinal flora burden per time.^13,16,17^ However, the effectiveness of the intestinal flora can be greatly influenced by purine-rich foods, which may disturb the purine metabolism process, thereby elevating serum UA levels.^18,19^ It is thus necessary to control UA from external sources to prevent hyperuricemia.^3^

Currently, several methods are used to treat hyperuricemia, including diet, drugs and biotherapy, aimed at purine uptake and degradation.^18,20,21^ An example is the representative UA-lowering drug, Allopurinol, which can competitively bind with the xanthine oxidase (XOD) enzyme to reduce UA production.^22^ Febuxostat is another novel non-purine selective inhibitor of XOD.^23^ Both drugs are widely used to treat hyperuricemia and they are relative safety.^24^ Dietary interventions work by restricting the intake of high-purine foods and alcohol, but can be less effective than drugs because of the difficulty in adhering to dietary restrictions for a long time. Microbial remediation as a cost-effective treatment approach has been greatly accepted by the public due to its minimal site disruptions in the body.^25^ Thus, several *Lactobacillus* strains that can utilize purines have been discovered. Among these are the strains found to be effective in lowering UA in rodents. *L. brevis* (DM9218) and *L. gasseri* (PA-3) for instance degraded the intermediate products of purine metabolism to ameliorate hyperuricemia.^26,27^ For literature, most of these *Lactobacillus* strains have also been aimed at intestinal degradation and absorption of purines.

Fermented foods are rich in lactic acid bacteria, and can produce organic acids to control decaying microorganisms and pathogens.^28,29^ Research has shown that fermented foods such as yogurt are beneficial for type 2 diabetes patients, because of its richness in lactic acid bacteria.^30^ Probiotics have also been reported to be effective in degrading UA, so we hypothesized that the activity of human intestinal flora can be enhanced by probiotics.^10,31^ “Jiangshui” is a traditional fermented food in Northwestern China, which is made with vegetables, such as celery and cabbage. Lactic acid bacteria, acetic acid bacteria and yeast are the main microbes in the Jiangshui fermentation process, especially the facultative anaerobic lactic acid bacteria.^32^ Thus, the present study is aimed at screening microbial strains from fermented Jiangshui with UA degradation ability to evaluate the effects of these strains on UA-induced hyperuricemia in a mouse model.

## 2. Result:

### 2.1. Correlation between the frequency of Jiangshui noodle and Gout

To explore the correlation between the consumption of Jiangshui and the incidence of gout, a questionnaire survey was conducted on 180 residents in Lanzhou city. The results showed that when the consumption frequency of Jiangshui noodle exceeded six times a week, the prevalence of gout displayed a downtrend (Supplementary File 2, Figure 1a). Also, Pearson’s analysis indicated a negative correlation between Jiangshui noodle and gout (Supplementary File 2, Figure 1b), which suggested that people who regularly ate Jiangshui noodles were less likely to develop gout.

**Figure 1.**
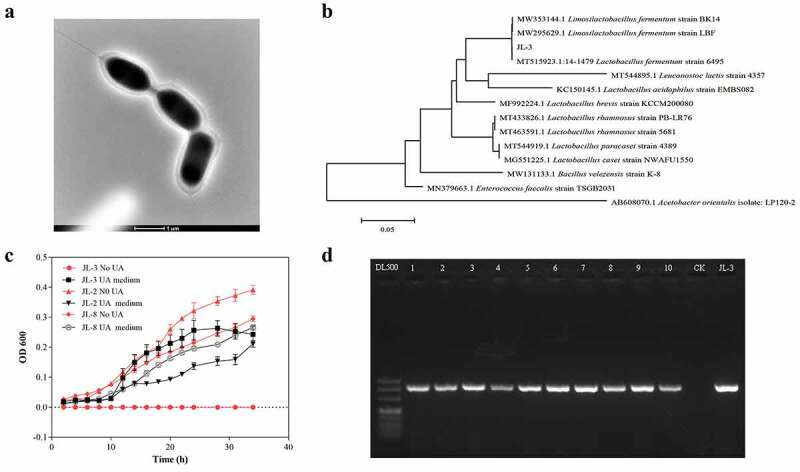
**Screening and identification of L-3 strain in Jiangshui. A]** Scanning electron microscope morphology of JL-3. **B]** Phylogenetic tree of strain JL-3 and related bacteria. **C]** The effects of different media on the growth of JL-2, JL-3, and JL-8. Compared with JL-2 and JL-8, JL-3 had the best growth effect in the medium containing UA, and there was no growth phenomenon in the medium without UA. **D]** Identification of JL-3 in 10 kinds of Jiangshui bought from Lanzhou supermarket (Gansu, China)

### 2.2. Isolation of UA degrading strains and product identification

To explore whether probiotics in Jiangshui had beneficial effects on gout, twenty strains with UA resistance activity were isolated from commercially fermented Jiangshui and cultured in a medium containing UA as the sole carbon and nitrogen source. Three strains JL-2, JL-3 and JL-8 could survive, of which the growth rate of JL-3 was significantly higher than the other two strains, showing an OD_600_ value above 0.25 within a 24 h of incubation period (Figure 1c). When compared with the NCBI BLAST database, the JL-3 strain was closely related to *Limosilactobacillus fermentum*^33^ (Figure 1b). Transmission election microscopy (TEM) also showed that JL-3 had a typical morphology of *Limosilactobacillus bacilli* (Figure 1a).

**Figure 2.**
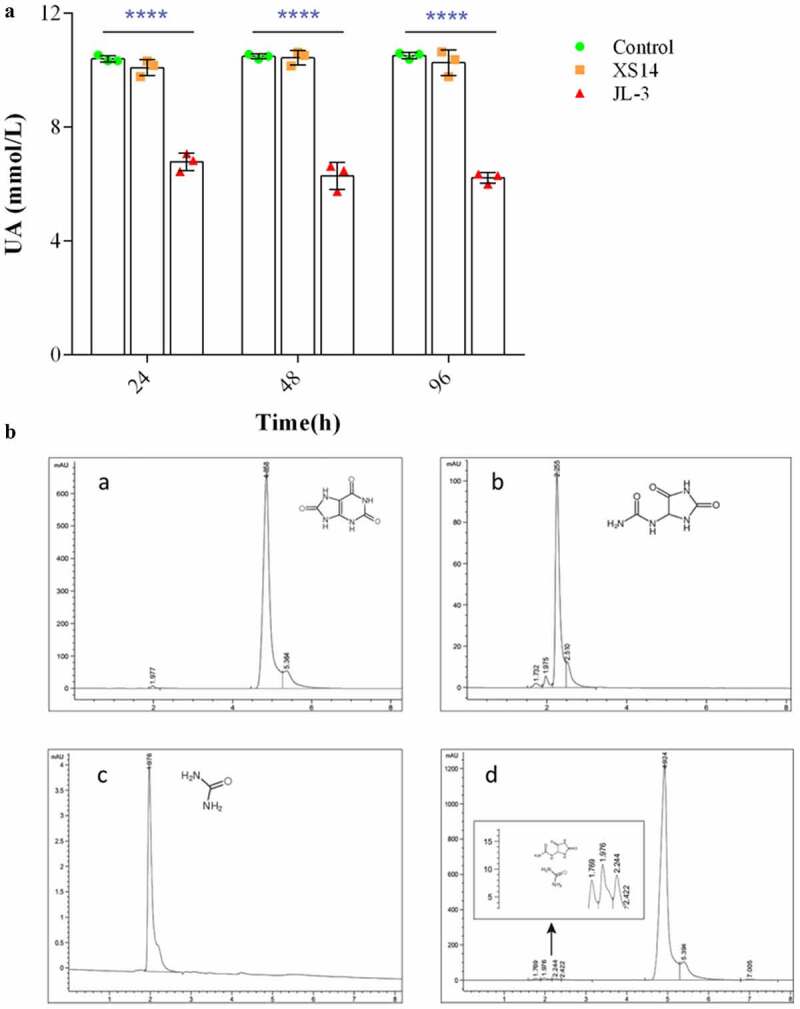
**Detection of UA degradation products by JL-3. A]** The UA degradation ability of the JL-3 strain *in-vitro*. **B]** The UA degradation products of JL-3 detected by HPLC. A. Detection peak of UA standard; b. Detection peak of allantoin standard; c. Detection peak of urea standard; d. The main metabolism products of JL-3 were urea and allantoin. Bars showed the mean ± SD (n = 5 mice per group) **P < .01; ****P < .0001

To analyze the UA degrading ability of JL-3, it was added to a sterile phosphate-buffered saline (PBS) solution containing UA (10 mmol/L) for cultivation. Results showed that the JL-3 strain could degrade 40.9% of UA within 24 h, compared to XS-14 which is a *limosilactobacillus fermentum* strain from yogurt (Figure 2a). Alternatively, high power liquid chromatography (HPLC) results revealed absorption peaks of UA and that of its degradation products at 4.868, 2.255 and 1.976 respectively, which was consistent with the standard of UA, allantoin and urea peaks (Figure 2b). This suggested that JL-3 could break down UA into smaller molecules such as allantoin and urea. Simultaneously, polymerase chain reaction (PCR) results of different commercial Jiangshui showed that JL-3 is widely present in various Jiangshui samples (Figure 1d).

### 2.3. JL-3 decreases UA level in hyperuricemic mice

**Figure 3.**
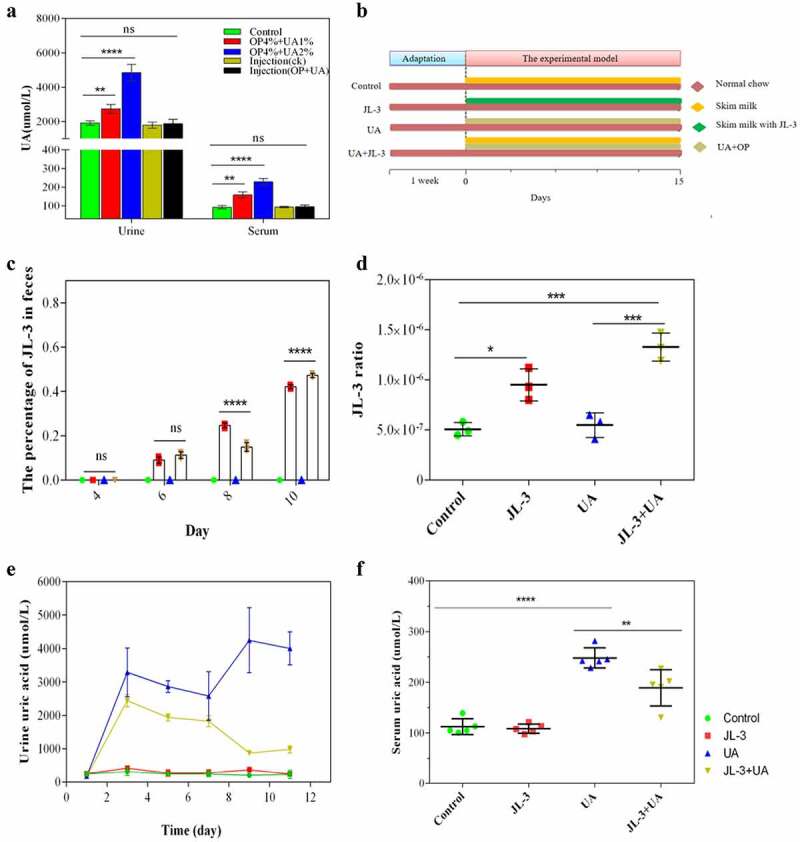
**Effect of JL-3 on hyperuricemia mice. A]** The hyperuricemia mouse model was established by detecting UA in urine and serum. In 2% concentration of UA and 4% of OP group, the UA level was significantly high, which was selected for experiment on JL-3 treatment. **B]** Experimental chart of JL-3 in treatment of hyperuricemia mice. **C]** Detection of JL-3 colonization by PCR at 4, 6, 8, and 10 d. **D]** The proportion of JL-3 in fecal flora determined by RT-PCR on day 22. **E]** The content of UA in the urine after 0, 2, 4, 6, 8, 10, and 12 d. **F]** The serum UA of mice after the experiment. Bars show the mean ± SD (n = 5 mice per group) **P < .01; **** *P* < .0001

The previous experiment proved that JL-3 could degrade UA. To test its ability in mice, the hyperuricemia mice model was constructed (Figure 3a). The constructed model of the hyperuricemia mice efficiently elevated serum UA in mice more stably, compared with oteracil potassium or high purine diet alone to sustain a longer duration of hyperuricemia.^26,34,35^ Thus, this method was adopted to construct hyperuricemia mice and gavaged with the strain (Figure 3b). Feces of mice were collected to investigate the survival of JL-3. The relative abundance of JL-3 strain in the feces of mice supplemented with this strain was significantly higher than the control group from day six as detected by PCR (Figure 3c). On day 14, QRT-PCR detection showed that the rate of JL-3 had reached 10^−6^ (*P* < .001) (Figure 3d). Further analysis also showed that compared with the UA group, the colonization of JL-3 reduced UA content in the blood and urine of mice. From day one to day three, UA levels of the UA group and JL-3 + UA group both increased rapidly, reaching more than 15 times that of the control group (*P* < .0001). However, UA levels of the JL-3 + UA group in urine gradually decreased from day three, and approached the control level on day nine. In serum samples collected on day 15, UA levels of the UA group (249.9 µmol/L) were 2.2 times higher than the control (112.6 µmol/L). After intervention with JL-3, UA concentrations decreased by about 31.3% compared with the UA group (*P* < .01) (Figure 3e). Compared with the control group, there was no significant difference between UA levels of urine and blood samples from the JL-3 group, which demonstrated that JL-3 could effectively reduce UA in hyperuricemia mice (figure 3f).

### 2.4. JL-3 strain attenuated UA-induced oxidative stress and inflammation

**Figure 4.**
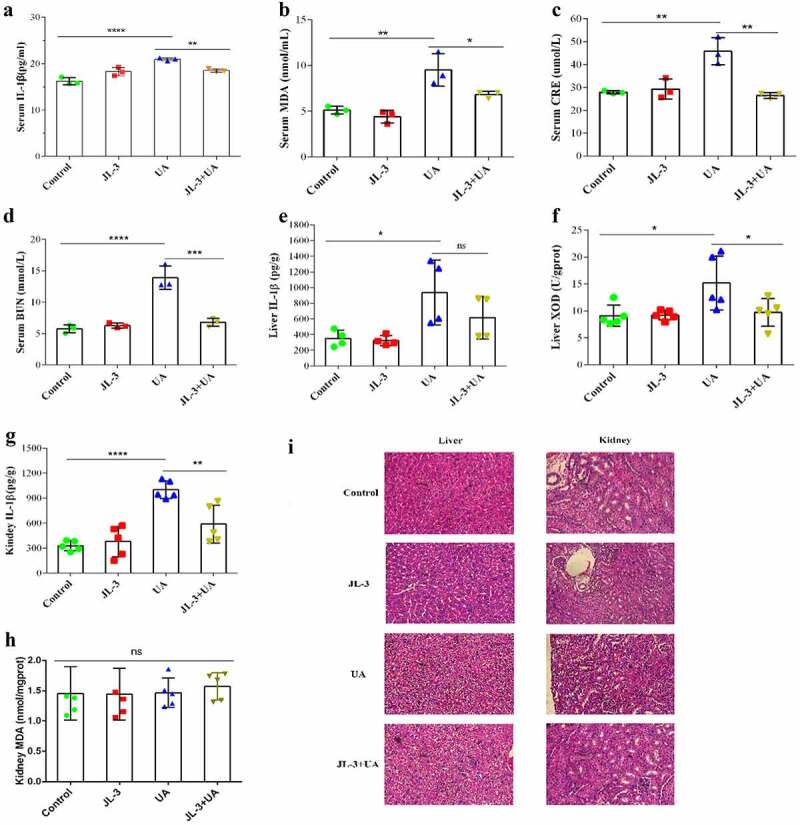
**Inflammation markers and oxidative stress indicators on hyperuricemia mice processed by JL-3**. Levels of interleukin-1β (IL-1β) (a), MDA (b), CRE (c), BUN (d) in mice serum in four groups. **E, F]** Levels of interleukin-1β (IL-1β) and MDA in mice liver. **G, H]** Levels of interleukin-1β (IL-1β) and the content of XOD in mice kidneys in different groups. **I]** Representative photomicrographs of H&E staining of mouse liver tissue. Mice were killed after feeding for 15 d, and serum was harvested from whole blood. Bars show the mean ± SD (n = 5 mice per group). **P < .01; ****P < .0001

**Figure 5.**
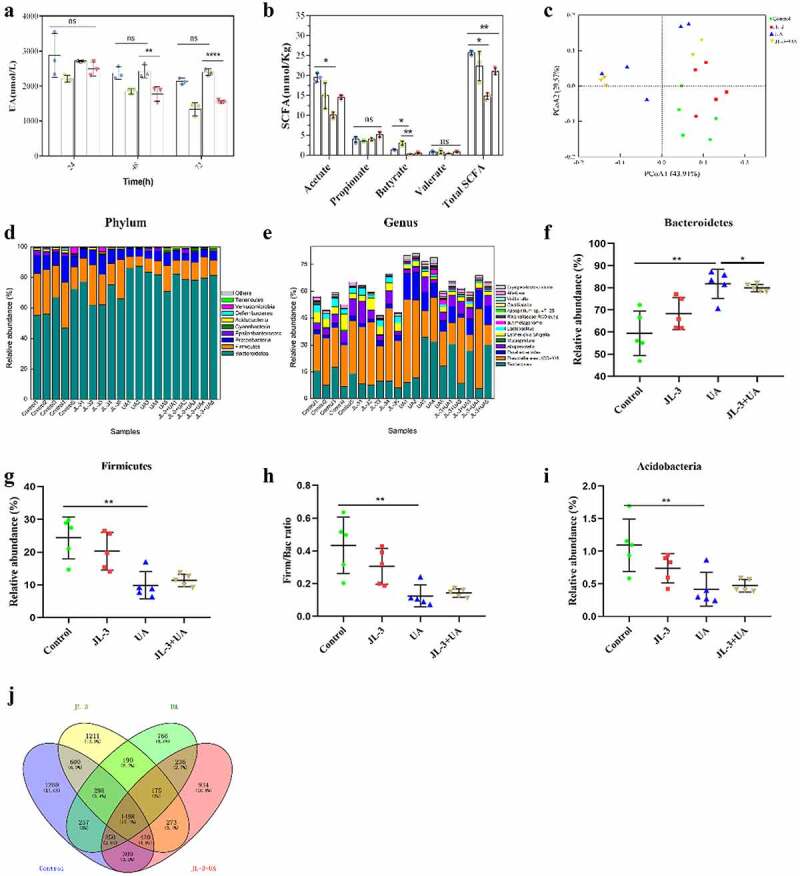
**Effects of JL-3 treatment on the function and microbial diversity of the gut microbiota in UA-exposed mice. A]** Degradation of UA by fecal flora of mice in different groups. **B]** The concentration of short-chain fatty acids in mouse feces. **C]** Principle-coordinates analysis (PCoA) of the overall diversity based on the Bray-Curtis distances. Scatter plot of PCoA scores depicting variances derived from bacterial communities in the four groups. **D]** Comparison of phylum relative abundance in different groups. **E]** The relative abundance of the most abundant bacterial genera in the four groups. **F, G, I]** Relative abundances of significantly changed bacterial phyla (*Bacteroidetes, Firmicutes*, and *Acidobacteria*). **H]** The Firm/Bac ratio in the different groups. **I]** Venn diagram analysis of OTU overlaps between different groups of microorganisms. The compared model group, it can modulate the intestinal flora structure when mice were given JL-3. Bars show the mean ± SD (n = 5 mice per group) **P < .01; **** *P* < .0001

To further analyze the effect of JL-3 on the physiological indexes of mice, the levels of IL-1β, malonaldehyde (MDA), creatinine (CRE) and blood urea nitrogen (BUN) were tested, which all showed an increasing trend in the UA mice group (*P* < .01) (Figure 4a, B, C, D). Also, when compared with the UA group, levels of these markers in the serum of the JL-3 + UA group were greatly reduced (*P* < .01). Specifically, IL-1β was reduced by 50.9%, BUN by 66.7% (accounting), MDA by about 40%, and CRE by 73%. In the kidney and liver, IL-1β level in the UA group was significantly increased *P* < .01). However, it partially dropped in the JL-3 + UA group (Figure 4e, G). Alternatively, xanthine oxidase (XOD) levels were increased in the liver of the UA group and restored to the control level after JL-3 intervention (figure 4f). Also, there was no significant difference in the kidney MDA level among the four groups (Figure 4h). Histopathological analysis showed that UA exposure induced liver damage, as liver sections from the UA mice group showed that cell space was increased, and nuclear staining deepened compared with the control mice. However, these symptoms were partially relieved in the JL-3+ UA group. However, they were no significant difference between the four groups in the kidney (Figure 5i).

### 2.5. JL-3 regulated UA-induced gut microbiota dysbiosis in hyperuricemic mice.

To investigate whether JL-3 could influence UA decomposition ability of the intestinal flora, we compared the UA degradation ability of feces bacteria in each group. As shown in Figure 5a, after 72 h of cultivation in UA medium, the feces supplemented with JL-3 mice degraded more than 55% of the initial UA in the medium (*P* < .0001). SCFAs detection by GC-MS in the feces of mice on day 15^th^, revealed that the total SCFAs and acetic acid concentrations in feces of the UA group were significantly lower than the control (*P* < .01). They were recovered in the JL-3+ UA group, but showed difference with the control group. In the JL-3 group mice feces, the butyric acid concentration also significantly increased compared with the control group (*P* < .01) (Figure 5b).

After that, we performed Miseq sequencing analysis of 16S rRNA to determine the diversity and abundance of gut microbiota associated with UA and/or *Limosilactobacillus fermentum JL-3* treated mice. At the end of the experiment, the abundance of microbial flora (Shannon index) was higher in UA+JL-3 group than the UA group, which was regulated by JL-3 treatment (Supplementary Table 1). Alternatively, Principle-coordinates analysis (PCoA) results showed that phylogenetic community structures were markedly different between UA-exposed samples and others, and that the UA group was separated from other groups. It suggested that UA exposure changed the gut microbiota composition, while JL-3 supplement diminished the effect of UA on microbiota changes (Figure 5c). The relative abundance at the phylum level of each individual sample in four groups was analyzed as shown in Figure 5d. The dominant phyla in the gut microbiota were *Bacteroidetes, Firmicutes, Proteobacteria, Epsilonbacteraeota* and *Acidobacteria* (relative abundance > 0.5%). Comparing the UA mice group to the control, there was a significant increase in the relative abundance of *Bacteroidetes* and a reduction in *Firmicutes* and *Proteobacteria*. JL-3 treatment attenuated UA-induced increase in *Bacteroidetes* and reduction in *Firmicutes* and *Acidobacteria* (figure 5f, G, I). The ratio of *Firmicutes* to *Bacteroidetes* (Firm/Bac ratio) was also deceased on UA exposure mitigated by JL-3 treatment (Figure 5h). At the genus level (Figure 5e), results showed that the relative abundance of *Erysipelatoclostridium and Mucispirillum* increased after UA exposure, while that of *Alloprevotella* and *Oscillibacter* decreased. JL-3 administrations countered UA-induced changes of the intestinal flora as well. In the JL-3+ UA group, *Alloprevotella* and *Oscillibacter* levels were higher than the UA group by 107.15% and 125.22%, respectively. The abundance of *Erysipelatoclostridium* and *Mucispirillum*, 64.01% and 65.65% respectively, were also less than that of the UA treated group, which had high levels in the diseased organism’s body.^36^ Venn analysis also showed that UA treatment significantly affected the structure of the intestinal flora (Figure 5j). All these results indicated that JL-3 has a positive effect on gut microbiota.

## 3. Discussion and Conclusion

### 3.1. Discussion

Hyperuricemia continues to pose health problems around the world.^37^ Although its prevalence is influenced by genetic factors, alcohol consumption, obesity, and hypertension are important factors that partially account for the recorded UA burden per time.^38^ Gut remediation is a simple, economic, and effective measure for humans by introducing specific probiotics as dietary supplements.^25,39^ Thus, fermented foods rich in bacteria have been reported effective against many diseases.^26^ Previous studies have authenticated the effects of fermented foods in effectively relieving diseases through their antioxidant activity *in-vivo*.^40,41^ Kimchi has shown an antioxidant effect in the prevention of atherosclerosis.^42^ Cheese lowers the risk of cardiovascular diseases by 2%.^43^ Meanwhile, previous research also proved that lactic acid bacteria in fermented foods have protective effects against some heavy metals and antibiotic residues in mammals.^25,44^ Scientific reports have also shown that Jiangshui noodles are rich in lactic acid bacteria.^32^ Furthermore, a survey reported that the incidence of gout in northern areas including Gansu Province was approximately 0.5% lower than the national average level (1.1%), which was coincident with our results.^45^ Studies have also shown that Jiangshui has a high degrading capacity of 75%.^46^ Therefore, we proposed that the effect on gout is due to the action of probiotics present in fermented Jiangshui.

This study focused on probiotic treatment for hyperuricemia, while earlier studies mainly concentrated on the purine metabolism, and only a few focus on UA.^18,19,47^ UA has been established as the major etiologic factor in hyperuricemia.^48^ Research has also found that it can be used as a nitrogen source by many aerobic organisms, such as *Escherichia coli*. The general catabolic pathway of UA by bacteria is allantoin, which proceeds via three steps, involving the interconversion between 5-hydroxyisouric acid, 2-oxo-4-hydroxy-4-carboxy-5-ureidoimidazoline and allantoin, which could easily degrade to ammonia.^49,50^ In our study, allantoin was detected as a by-product of JL-3, which is coincident with former reports, as its degradation rate was found to be more significant compared with *Bacteroides termitidis* UAD-50 (50 µmol/2 h).^47,51^

After 15 d of intervention, the amount of JL-3 in the feces exceeded 10^6^ CFU/g, which was in accordance with a previous study, thus indicating that the JL-3 strain continued to survive in the mouse intestine throughout the experiment.^52^ The beneficial effect of JL-3 on hyperuricemia mice was evidenced by the decreased UA levels, as results showed that the UA levels in JL-3 + UA group significantly decreased to more than 30% in feces and urine compared with the UA group. This was effectively reduced compared with DM9218 (< 30%) and the microbial fermented extracts of *Lactobacillus* (21.6%).^26,53^ Consistent with previous studies, we found a dramatic increase in BUN and CRE after UA injection, which is associated with hyperuricemia, chronic nephritis and renal insufficiency.^54,55^ In addition, serum MDA was also increased, as was the IL-1β in the liver and kidney, which is similar to the high-fructose diet.^56^ However, after JL-3 treatment, the levels of all these parameters were modulated to normal. XOD catalyzes the hydroxylation of hypoxanthine and xanthine, which are the last two steps of urate biosynthesis.^57^ After intervention with JL-3, increased XOD activity in the liver was restored to normal, suggesting that JL-3 inhibited XOD. Similar XOD-inhibiting effects were reported in microbial fermented extracts of *Aspergillus* fungi (40.8% – 63.5%), but the underlying mechanism is unclear and deserves further investigation. This suggests that UA-induced oxidative stress and inflammatory response in the liver. The over production of XOD has been demonstrated to cause an increased production of UA to further increase inflammation. In addition, consistent with previous research on *L. acidophilus* KB27 and *L. rhamnosus* KB79^58^, H&E photomicrographs further showed the characteristics of injury in hyperuricemia mice kidney that was significantly reduced after the JL-3 intervention.^58,59^

Specifically, the UA degradation capacity of fecal bacteria in UA + JL-3 mice was remarkably higher than that in mice treated solely with UA. This suggested that the UA-degradation ability of fecal bacteria in the UA + JL-3 group was enhanced by the addition of JL-3. The restorative effect of JL-3 on the metabolism of gut microbiota was also evidenced by the recovery of intestinal SCFAs. Studies have shown that SCFAs are important energy and signaling molecules produced by intestinal microorganisms associated with gout. Similar to the acetic acid in the intestine, SCFAs can serve as a central regulator for the intensity and duration of gout inflammation.^60,61^ This revealed that JL-3 could restore intestinal metabolism and promote UA degradation.

Gut microbiota is characterized by temporal stability and resilience.^62^ Studies have demonstrated that the gut microbiota might be related to gut inflammatory responses, which participate in a series of diseases.^63^ However, many external factors that exceed the tolerance capacity of the gut microbiota can lead to dysbiosis.^25^ As an antioxidant and immune enhancer, UA exerts a great impact on the gut microbiota community.^64^ UA exposure significantly reduced the diversity and abundance of gut microbiota compared to those of the control group mice, which was regulated by JL-3 treatment, suggesting that JL-3 improves the gut microbiota disturbed by UA exposure. At the phylum level, UA significantly increased the relative abundance of *Bacteroidetes*, but decreased that of *Firmicutes* and *Acidobacteria*, which impart key functions to their host, such as metabolic, developmental, and immunologic properties.^65–67^ This is similar to a previous research based on the Sprague–Dawley rats with hyperuricemia.^57^ However, JL-3 pretreatment significantly attenuated UA induced increase in *Bacteroidetes* and decrease in *Firmicutes* and *Acidobacteria*. At the genus level, *Prevotella* and *Bacteroidales* were negatively associated with testicular function, while *Paraprevotella, Mucispirillum, Oscillospira, Coprococcus* and *Lactobacillus* were positively correlated with that function.^63,68^ JL-3 pretreatment reduced the increase of *Prevotella* and *Bacteroidales*, and elevated the decrease of *Paraprevotella, Mucispirillum, Oscillospira* and *Lactobacillus* in UA-exposed mice by regulating intestinal flora and improving intestinal function to alleviate gout.^62,63^ In addition, the low abundance inherent strain of *Erysipelatoclostridium* that appears in a host colitis by competing for nutrients negatively affected the flora.^69,70^ In correspondence with our reports, *L. acidophilus* KB27 and *L. rhamnosus* KB79 supplements also confirmed the fact that mice gut microbiota were rich in *Firmicutes* and *Bacteroidetes*.^58^ Thus, it can be concluded that the feed addition of JL-3 can reduce the phylum level disturbance associated with hyperuricemia and maintain the steady-state of the mice intestinal microbial community.

Based on these experimental results, it is hypothesized that JL-3 might exert its effects in multiple ways. First, JL-3 can degrade UA in the gut, thus reducing the amount of UA that accumulates in the intestinal tract, thereby lessening the amount of UA reabsorbed by the intestinal epithelium reentering circulation. The protective mechanism of JL-3 against UA accumulation in mice was also found to be similar to several reports on purine-degrading *Lactobacillus* strains.^25,52,71^ Second, JL-3 can improve bowel movement activity, thus decreasing fecal excretion of UA. Thirdly, JL-3 can modulate the structure and function of gut microbiota.

### 3.2. Conclusion

In this study, *Limosilactobacillus fermentum* JL-3, a candidate probiotic strain was derived from fermented dairy products with UA reduction capability. This study explored the degradation of UA by JL-3 using mice as an animal model. The bacteria administered to mice, colonized the intestine, while JL-3 decreased UA levels in mice, and regulated the structure and function of the gut microbial community. Compared to current treatment technologies, probiotics appear to be direct and efficient at repairing hyperuricemia, thus playing an important role in hyperuricemia treatment in humans, which might provide a promising strategy for alleviating hyperuricemia.

## 4. Materials and methods

### 4.1. Questionnaire on “Jiangshui” noodle consumption

A cross-sectional comparison of the questionnaire with a standard chart abstraction-based measure (Pearson Index) was conducted on volunteers among the population in Gansu Province (Supplementary File1). The questionnaire survey was conducted anonymously to count the gout conditions, and statistically investigate the incidence of related behaviors. The prevalence of gout was investigated in 180 residents with eight questions regarding gout. The survey mainly focused on: 1) the frequency of Jiangshui noodle consumption every week; 2) the situation of gout; 3) the frequency of dietary habits such as drinking and eating meat. SPSS v.17.0 software was used to analyze the data at *P* < 0.05. The difference was statistically significant.

### 4.2. Isolation of the bacterial strain with UA degrading ability

Traditional Jiangshui samples were collected from Lanzhou supermarket (Gansu, China). From the collected samples, 20 strains were isolated in a UA medium (1.71 g Na_2_HPO_4_. 12 H_2_O, 0.3 g KH_2_PO_4_, 0.05 g NaCl, 0.05 g MgSO_4_.7 H_2_O, 0.001 g CaCl_2_, 0.2 g UA and 1.2 g agar, per 100mL) (Supplementary Table 2).^72^ The selected strains were then transferred to a new medium with UA as the sole carbon source. The grown bacterial culture plates were kept in an incubator at 37°C for 48 h. While the obtained bacterial colonies (JL-2, JL-3 and JL-8) were further streaked on new MRS agar plates to acquire a single colony. The new monoclonal colony activated in MRS medium was then stored in 25% glycerol at −80°C.

### 4.3. Determination of uric acid degradation ability of strains in vitro

To evaluate the UA degradation ability, JL-2, JL-3 and JL-8 were inoculated onto MRS and cultured for 48 h at 37°C under anaerobic conditions. 2 mL of the culture solution was centrifuged at 4,000 ×*g* for 10 min, after which the cells were washed twice with 1 mL 0.85% NaCl, resuspended in 750 mL of UA solution and incubated at 37°C for 60 min. Later, the solution was centrifuged at 4,000 ×*g* for 10 min to obtain the supernatant. Thirty milliliters HClO_4_ (0.1 mol/L) was then added to the supernatant and mixed thoroughly to prevent further degradation, after which 20 mL of the mixture was injected into the HPLC device after filtration. The remaining urea and allantoin contents were calculated. The degradation rate of JL-3 strains was also calculated according to the following formula: V = (0.9 C − X)/60, a = [(0.9 C − X)/0.9 C] N100%.

Where V: degrading speed (g/L/min), X: the remaining content of urea or allantoin (g/L).^73,74^ Adenylate-guanylate-neutral potassium phosphate culture solution (900 μL) was mixed with 100 μL of terminator perchloric acid solution. After mixing, 20 μL was aspirated. The external standard method was then used to determine the retention time of UA to generate a quantitative standard curve. The conditions for HPLC were as follows: Water XTERRA MS C18 (250 mm × 4.6 μM), the mobile phase was a gradient of 20 mmol/L potassium dihydrogen phosphate solution (pH 5.0), with a flow rate of 1 mL/min, while column temperature was 25^°^C, with wavelengths of 254 nm (UA), 190 nm (urea), 190 nm (allantoin), and an elution time of 40 min.

## 4.4. 16S rRNA gene sequencing and phylogenetic analysis

TIANamp Bacteria DNA Kit (TIANGEN Biotechnology, China) was used to obtain high quality genomic DNA of strains. The 16S rDNA was amplified as described by Sibley *et al*.^75^ The sequence of potential isolates for species identification was made using the BLAST engine (NCBI) and the nucleotide sequence was submitted to GenBank for reference (SUB8885938 SEQ1 MW474833, SUB8885938 SEQ2 MW474834, SUB8885938 SEQ3 MW474835).^76^ The morphology of JL-3 was analyzed by scanning electron microscopy (SEM S-3400 N II Hitachi, Japan), while the multiple sequences of JL-3 and other closely related strains were aligned by MEGA (Molecular Evolutionary Genetics Analysis, v.6.0).^77,78^

### 4.5. Experimental design based on the animal model

Fifty Kunming male mice (Lanzhou University Medical Animal Testing Center) which were 4-week-old and weighed 16–20 g, were bought. Before the experiment, mice were habituated for a week. For the experimental procedures, 1 mouse per cage was housed at 22°C ± 1°C and 60%–75% relative humidity. The mice were then fed with standard commercial mouse food. The care and use of laboratory animals in our study was approved by the Lanzhou University Animal Ethics Committee, after that the relevant ethical regulations were followed.

For inducting of UA-induced hyperuricemia, mice were given oteracil potassium (OP) and UA in 0.5 mL water for 14 d. Fifty mice were randomly divided into five groups, where OP and UA were used to construct the hyperuricemia animal model according to literature.^34,79^ The control group was then fed with a basal diet, and orally gavaged with 0.5 mL of water, while the UA_1_ group was given the basal diet along with OP (4%) and UA (1%) injection through the oral gavaged route. The UA_2_ group was also given the basal diet of OP (4 %) and UA (2 %), which were injected through the oral gavaged route, while the UA_3_ group was fed the basal diet and intraperitoneally injected with OP (4%) and UA (2%) in 0.5 mL water. The blank group was then fed with a basal diet and intraperitoneal injection of 0.5 mL water. During this period, urine and blood samples were collected to determine whether the model was well established by detecting the UA content. According to an earlier research, different concentrations of UA were selected to feed mice by injection to establish hyperuricemia model mice.^34^

The 2% concentration of UA and 4% of OP confirmed during the model experiment were used for repair experiments. Forty mice were randomly allocated into four groups (10 mice per group). The control group was fed with the basal diet and sterile skim milk (0.5 mL) administrated by oral gavage every day, while the JL-3 group was also fed with a basal diet and gavage with JL-3 in the sterile skim milk. The UA group, on the other hand, was fed with basal diet plus 2% UA and 4% OP, and orally gavaged sterile skim milk (0.5 mL), while the UA + JL-3 group received a basal diet containing 2% UA and 4% OP, while being orally gavaged with the JL-3 strain in sterile skim milk. About 1×10^8^ CFU of JL-3 was dissolved into 0.5 mL of skim milk, which was directly injected into the mouth of each mouse twice a day (9:00 in the morning and 18:00 in the afternoon). Skim milk was prepared using sterilizing skimmed milk powder at 15 min, while 1 mL of bacterial solution was centrifuged at 8,000 ×*g* for 10 min, after which the suspension of the sterilized milk was used.

During the 15 d of treatment, all mice in each group were moved into a clean and empty cage every three days for an hour, and fecal and urine samples were collected. Later, the mice were anesthetized using diethyl ether. Blood was collected from the eye vein and was left undisturbed for 30 min. The collected blood samples were centrifuged at 4000 rpm for 20 min to get the serum. Also, liver, kidney and intestine samples were gathered for the respective groups. The collected samples were then washed with saline, collected in pre-weighted tubes with 1 mL PBS diluent, and stored at −20°C for future use. All samples were preserved at −80°C until analysis, except those used for histopathology, which were stored in 4% paraformaldehyde.

### 4.6. *Colonization of* Limosilactobacillus fermentum *JL-3*

During the experiment, mice feces were collected after 4, 6, 8, and 10 d. PCR was then performed using specific primers of the *Limosilactobacillus fermentum* as reported in previous literatures.^80,81^ All primer sequences for qPCR are listed: specific primers of JL-3 (F: ACGTATGAACAGTTACTCTCATACGT; R: CCTGATTGATTTGGTCGCCAAC; and general primers       of      bacteria        (F-tot: GCAGGCCTAACACATGCAAGTC;  R-tot:CTGCTGCCTCCCGTAGGAGT), were used to obtain PCR products. Then, semi-quantitative fluorescence analysis of JL-3 was performed using Image J-win v.32.

On the 22nd day, fresh mouse feces were cultured in the MRS medium, and incubated for 48 h at 37°C in a static incubator, then sub-cultured again. Mouse fecal DNA was then extracted according to the manufacturer’s instructions using TIANamp Stool DNA Kit. To quantify the total bacteria in the feces samples, primers tot-F and tot-R were used for amplifying the 16S rDNA, while was run on a Bio-RAD CFX96 (USA) in a total volume of 10 μL, using SYBR Premix Ex Taq II (Takara) on real-time quantification PCR instrument (Bio-RAD CFX96, USA). All measurements were performed in triplicate. Standard curves were then constructed using PCR products of the 16S rRNA gene of JL-3 and *E. coli*, after which the obtained PCR products were cloned into T vector (Takara, Dalian, China) and transfected into DH-5α, which was then incubated at 37°C for 6 h (100 −140 rpm) by Plasmid Mini Kit I (E.Z.N.A., Omega, USA) as the standard sample. The recombinant plasmids were used for qPCR, with 10^3^, 10^4^, 10^5^, 10^6^, 10^7^, 10^8^, 10^9^, and 10^10^ copies of the plasmids per reaction used for calibration. The target copy numbers (T) were then estimated using the equation: T = (D/(PL × 660)) × 6.022 × 1,023,133, Where D (g/l) and PL (base pairs) were the plasmid DNA concentrations and lengths, respectively. Each standard curve was then generated from at least five 10-fold plasmid dilutions in triplicate. The proportion of JL-3 in the whole bacterial community of bacteria in different samples was calculated based on the standard curve.^82,83^

### 4.7. Determination of UA in mice

The frozen mouse feces in 1 ml PBS were placed in a 60°C water bath for 10 min, and vortexed for 1 min to break up the stool sample. The solution was collected by centrifugation at 1000 rpm for 5 min. Fresh mouse urine was placed in 60°C water bath and diluted 10 times with distilled water to ensure that no urate deposits were present in the urine sample, after which all samples were strictly detected according to the instructions provided in the UA detection kit (JianchengNanjing, China). This kit provides a fluorescence-based method for detecting UA. In the assay, uricase transforms UA to allantoin, hydrogen peroxide (H_2_O_2_), and carbon dioxide. In the presence of horseradish peroxidase, H_2_O_2_ reacts with 10- acetyl-3, 7-dihydroxyphenoxazine to produce the highly fluorescent compound resorufin. Resorufin fluorescence was then analyzed at an excitation wavelength of 530 nm and an emission wavelength of 595 nm, after which the concentration of UA in the serum and urine samples were calculated using the equation determined from different doses of standards.^53^

### 4.8. Determination of short-chain fatty acid (SCFA) in mouse feces

The fecal samples were frozen immediately after collection and stored at −20°C until analysis, while 1g of the feces sample was thawed and suspended in at least 5 mL water and homogenized for about 3 min, resulting in a 17% (w/w) fecal suspension. The pH of the suspension was then adjusted to 2–3 by adding 5 M HCl, and then kept at room temperature for 10 min under occasional shaking. The suspension was then transferred into a polypropylene tube and centrifuged for 20 min at 5000 rpm, to give a clear supernatant, while the internal standard, 2-ethylbutyric acid solution (2EB), was spiked into the supernatant at a final concentration of 1 mm and injected in the GC for analysis. The response linearity for fecal samples was tested with the serial solutions prepared by diluting the above serial standard solutions with equal volume of fecal supernatant, after which the concentration of SCFAs were measured by GC-MS chromatographic workstation. N2 was used as the carrier. The concentration of SCFAs in the supernatant was detected by chromatographic column (FFAP 30 m × 0.32 mm × 0.5 µM) and FID detector, and the properties of SCFAs were determined afterward. The content of SCFAs in each sample was then calculated by comparing with the internal standard 2EB.^84^

### 4.9. Tissues collection and biomarker measurements

Fresh liver and kidneys were ground using a handheld grinder (Jingxin, Shanghai, China) on ice, after which the suspension of samples was obtained at low speeds centrifugation (8000 rpm, 10 min). The levels of XOD and IL-1β in the liver, MDA and IL-1β in kidney, and IL-1β, MDA, creatinine (CRE), and urea nitrogen (BUN) in serum samples were all then detected using commercial kits (Jiancheng, Nanjing, China) according to the protocols provided. At least 5 samples from each group were selected for independent assay.

The liver and kidney samples were then rinsed with PBS, after which the liver was taken with a volume of 0.8 × 0.8 cm, while whole kidneys were employed. The samples were fixed with 4 % paraformaldehyde solution at room temperature for more than one day. Later, the samples were sent to the medical college of Lanzhou university for physiological sections and staining with hematoxylin and eosin (HE). The morphology of the sections and the accumulation of UA in mice renal tubules in different groups were observed under a microscope (OLYMPUS BX53, Japan) afterward.

### 4.10. Degradation ability of UA by mice fecal flora

Mice stool samples were collected at different time points. At first, 1 g of stool was cultured in the YCFA medium as reported in literature,^85^ and then incubated for 48 h at 37°C in an anaerobic incubator. The samples were then transferred to a new anaerobic incubator for two days. The cell content in the medium was detected using OD600(METASH UV-5100). The media contents were centrifuged at 8,000 × g for 10 min with an equivalent community to obtain cell pellets (Handheld tissue grinder, Jingxin MY-10, China). The obtained cell pellets were then washed twice with pure sterile PBS and resuspended in pure PBS solution containing UA. Supernatant of the solution was taken from the anaerobic incubator every three days. The UA detection kit (JianchengNanjing, China) was used to detect the UA concentration changes according to the direction of the manufacturer.^86^

### 4.11. DNA extraction and 16S rRNA amplification

The feces were collected from individually housed mice the day before gavage treatment initiation, and stored at −80°C until DNA collection. The stool sample pellets were weighed to 0.2 g and DNA, isolated in ddH_2_O using a modified TIANamp Stool DNA Kit (JianchengNanjing, China) protocol. The obtained DNA was then stored at −20°C until use. According to the selection of sequencing regions, diluted genomic DNA served as templates, with specific primers having barcode and high-fidelity enzymes (Takara, Dalian) to ensure PCR amplification efficiency and accuracy. PCR was performed for 16S v4-v5 region of bacteria using a primer pair 515F (5′-GTGYCAGCMGCCGCGGTAA-3′) and 806R (5′-GGACTACNVGGGTWTCTAA-3′).^87^ The PCR mixture (25 μl) contained 1x PCR buffer, 1.5 mM MgCl_2_, and deoxynucleoside triphosphate at 0.4 μM, each primer at 1.0 μM and 0.5 U of Ex Taq (TaKaRa, Dalian) and 10 ng soil genomic DNA. The PCR amplification protocol included initial denaturation at 94 °C for 3 min, followed by 30 cycles of 94°C for 40 s, 56°C for 60 s, and 72°C for 60 s, and a final extension at 72°C for 10 min. Two PCR reactions were conducted for each sample, and combined together after PCR amplification.^88^ DNA concentration and purity were measured by NanoDrop (Thermo Scientific, USA). The PCR product was detected by 1% agarose gel electrophoresis. The product was recovered from the target strip using the DNA Gel Extraction Kit (OMEGA), and the concentration and quality of the product were determined by Nanodrop. The same amounts of samples were mixed according to the concentration of PCR products. The library was constructed with TruSeq® DNA PCR-Free Sample Preparation Kit and quantified using Qubit and qPCR (PRJNA691734). Later, it was sequenced by an Illumina sequencing platform (Miseq PE250),^89^ and the resulting sequence read files using the QIIME2 pipeline (Quantitative Insights Into Microbial Ecology; http://qiime2.org) and its plugins. Paired-end sequence splicing was also performed using flash (v.1.2.7, http://ccb.jhu.edu/software/FLASH/).^90, 91^ The reads of each sample were spliced, and the spliced sequences were raw tags. Singleton was removed from OTUs (OTUs with only one sequence in all samples). The OTU representative sequences were annotated by QIIME2 software, using silva-132-99-515-909-nb-classifier.qza as a reference database. Alternatively, the chloroplast and mitochondrial sequences of eukaryotes were removed, and QIIME2 software used to analyze observed species, evenness, Shannon, Faith’s PD and other diversity indexes were calculated, and dilution curve was drawn afterward. According to OTU table and phylogenetic tree, UniFrac distance matrix was generated and weighted, after which the UniFrac PCoA map was drawn.^92^

### 4.12. Statistical analysis

Statistical analysis was performed using SPSS v.17.0 software. *P*-value < 0.05 indicated statistical significance. Data were analyzed using one-way analysis of variance (ANOVA). The significant differences between the two groups were analyzed using an independent samples T-test. To determine the composition of the bacterial community, PCoA was performed using Rversion v.3.3.2.

## Supplementary Material

Supplemental Material

## References

[1] Falasca GF. Metabolic diseases: gout. Clin Dermatol. 2006;24(6):498–18.doi:10.1016/j.clindermatol.2006.07.015. 17113968

[2] Chaves MM, Sinflorio DA, Thorstenberg ML, Martins MDA, Moreira-Souza ACA, Rangel TP, Silva CLM, Bellio M, Canetti C, Coutinho-Silva R, et al. Non-canonical NLRP3 inflammasome activation and IL-1β signaling are necessary to L. amazonensis control mediated by P2X7 receptor and leukotriene B4. PLoS Pathog. 2019;15(6):e1007887. doi:10.1371/journal.ppat.1007887. 31233552 PMC6622556

[3] Ebrahimpour-koujan S, Saneei P, Larijani B, Esmaillzadeh A. Consumption of sugar sweetened beverages and dietary fructose in relation to risk of gout and hyperuricemia: a systematic review and meta-analysis. Crit Rev Food Sci Nutr. 2016;213(1):1–10. doi:10.1080/10408398.2018.1503155. 30277800

[4] Chen‐Xu M, Yokose C, Rai SK, Pillinger MH, Choi HK. Contemporary prevalence of gout and hyperuricemia in the United States and decadal trends: the national health and nutrition examination survey, 2007–2016. Arthritis & Rheumatology. 2019;71(6):991–999. doi:10.1002/art.40807. 30618180 PMC6536335

[5] Zhou Z, Li Z, Wang C, Li X, Cheng X, Li C, Shi, Y. Common variants in the SLC28A2 gene are associated with serum uric acid level and hyperuricemia and gout in Han Chinese. Hereditas. 2019;156:4. 30679935 10.1186/s41065-018-0078-0 PMC6335706

[6] Mattiuzzi C, Lippi G. Recent updates on worldwide gout epidemiology. Clin Rheumatol. 2019;:39:1–3. 10.1007/s10067-019-04868-9 31836936

[7] 刘国信. 痛风患者降”酸”巧用组合拳. 心血管病防治知识 2018;000:16–18.

[8] Pedley AM, Benkovic SJ. A new view into the regulation of purine metabolism: the purinosome. Trends Biochem Sci. 2017;42:141–154. 28029518 10.1016/j.tibs.2016.09.009 PMC5272809

[9] Vischer E, Chargaff E. The separation and quantitative estimation of purines and pyrimidines in minute amount. Journal of Biological Chemistry. 1948;176:703–714. 18889926

[10] Wempe MF, Jutabha P, Quade B, Iwen TJ, Frick MM, Ross IR, Rice PJ, Anzai N, Endou H. Developing Potent Human Uric Acid Transporter 1 (hURAT1) Inhibitors. J Med Chem. 2011;54(8):2701–2713. doi:10.1021/jm1015022. 21449597 PMC3124071

[11] Anc S, Mab L, Dichi I. The uric acid metabolism pathway as a therapeutic target in hyperuricemia related to metabolic syndrome. Expert Opin Ther Targets. 2012;16(12):1175–1187. doi:10.1517/14728222.2012.723694. 23020656

[12] García-Arroyo FE, Gonzaga G, Muñoz-Jiménez I, Blas-Marron MG, Silverio O, Tapia E, Soto V, Ranganathan N, Ranganathan P, Vyas U, et al. Probiotic supplements prevented oxonic acid-induced hyperuricemia and renal damage. PLoS One. 2018;13(8):e0202901. doi:10.1371/journal.pone.0202901. 30142173 PMC6108486

[13] Sorensen LB, Levinson DJ. Origin and extrarenal elimination of uric acid in man. Nephron. 1975;14(1):7–20. doi:10.1159/000180432. 1124137

[14] Choi HK, Atkinson K, Karlson EW, Willett W, Curhan G. Purine-rich foods, dairy and protein intake, and the risk of gout in men. New England Journal of Medicine. 2004;350(11):1093–1103. doi:10.1056/NEJMoa035700. 15014182

[15] Chen M, Chen S, Hsu H, Chen Y, Chen K, Yech Y, Wang L, Chan H. Method of reducing levels of uric acid. Google Patents. 2016.

[16] Gvd V. Van der Drift C. Degradation of purines and pyrimidines by microorganisms. Bacteriol Rev. 1976;40(2):403. doi:10.1128/BR.40.2.403-468.1976. 786256 PMC413962

[17] Grassi D, Ferri L, Desideri G, Di Giosia P, Cheli P, Del Pinto R, Properzi G. Chronic hyperuricemia, uric acid deposit and cardiovascular risk. Curr Pharm Des. 2013;19(13):2432–2438. doi:10.2174/1381612811319130011. 23173592 PMC3606968

[18] Ogawa J. Analysis of microbial purine metabolism and its application for hyperuricemia prevention. Division of Applied Life Sciences, Graduate School of Agriculture, Kyoto University; 2006. p. 32–34.

[19] Wang H, Mei L, Deng Y, Liu Y, Wei X, Liu M, Zhou J, Ma H, Zheng P, Yuan J, et al. Lactobacillus brevis DM9218 ameliorates fructose-induced hyperuricemia through inosine degradation and manipulation of intestinal dysbiosis. Nutrition. 2019;62:63–73. doi:10.1016/j.nut.2018.11.018. 30852460

[20] Gliozzi M, Malara N, Muscoli S, Mollace V. The treatment of hyperuricemia. Int J Cardiol. 2013;19:2432–2438. doi:10.1016/j.ijcard.2015.08.087. 26320372

[21] Kerman DH. Endoscopic Delivery of Fecal Biotherapy in Inflammatory Bowel Disease. Gastrointestinal Endoscopy Clinics of North America. 2016;26(4):707–717. doi:10.1016/j.giec.2016.06.006. 27633598

[22] Ahn SO, Ohtomo S, Kiyokawa J, Nakagawa T, Yamane M, Lee KJ, Kim KH, Kim BH, Tanaka J, Kawabe Y, et al. Stronger uricosuric effects of the novel selective URAT1 inhibitor UR-1102 lowered plasma urate in tufted capuchin monkeys to a greater extent than benzbromarone. Journal of Pharmacology and Experimental Therapeutics. 2016;357(1):157–166. doi:10.1124/jpet.115.231647. 26907620

[23] Becker MA, Schumacher HR Jr., Wortmann RL, MacDonald PA, Eustace D, Palo WA, Streit J, Joseph-Ridge N. Febuxostat compared with allopurinol in patients with hyperuricemia and gout. N Engl J Med. 2005;353(23):2450–2461. doi:10.1056/NEJMoa050373. 16339094

[24] Weng S, Shu K, Tarng D, Cheng C, Chen C, Yu T, Chuang Y, Huang S, Wu M. Uric acid is highly associated with kidney allograft survival in a time-varying analysis; Transplantation proceedings: Elsevier:2014. 505–510. 10.1016/j.transproceed.2013.09.038 24656000

[25] Nishita M, Park S-Y, Nishio T, Kamizaki K, Wang Z, Tamada K, Takumi T, Hashimoto R, Otani H, Pazour GJ, et al. Ror2 signaling regulates Golgi structure and transport through IFT20 for tumor invasiveness. Sci Rep. 2017;7(1):1–12. doi:10.1038/s41598-016-0028-x. 28127051 PMC5428335

[26] Li M, Yang D, Mei L, Yuan L, Xie A, Yuan J. Screening and Characterization of Purine Nucleoside Degrading Lactic Acid Bacteria Isolated from Chinese Sauerkraut and Evaluation of the Serum Uric Acid Lowering Effect in Hyperuricemic Rats. Plos One. 2014;9. 10.1371/journal.pone.0105577 PMC4153548 25184445

[27] Naruomi Y, Chizuru SI, Marie N, Misato S, Yoshika C, Hiroshi K, Yukio A. Lactobacillus gasseri PA-3 Uses the Purines IMP, Inosine and Hypoxanthine and Reduces their Absorption in Rats. Microorganisms. 2017;5(1):10. doi:10.3390/microorganisms5010010. PMC5374387 28282902

[28] Borresen EC, Henderson AJ, Kumar A, Weir TL, Ryan EP. Fermented foods: patented approaches and formulations for nutritional supplementation and health promotion. Recent Pat Food Nutr Agric. 2012;4(2):134–140. doi:10.2174/2212798411204020134. 22702745 PMC5175401

[29] Rhee SJ, Lee J-E, Lee C-H. Importance of lactic acid bacteria in Asian fermented foods. Microb Cell Fact. 2011;10(Suppl 1):S5. doi:10.1186/1475-2859-10-S1-S5. 21995342 PMC3231931

[30] Gille D, Schmid A, Walther B, Vergères VG. Fermented Food and Non-Communicable Chronic Diseases: a Review. Nutrients. 2018;10(4):10. doi:10.3390/nu10040448. PMC5946233 29617330

[31] Cao T, Li X, Mao T, Liu H, Zhao Q, Ding X, Li C, Zhang L, Tian Z. Probiotic therapy alleviates hyperuricemia in C57BL/6 mouse model. 2017.

[32] Xian-Gang M, Xue-Ping LI, Jian-Hong LI, Jia-Jia X, Nan Z. Isolation and identification of lactic acid bacteria from Jiangshui and preliminary study on metabolic characteristics. Sci Tech Food Industry. 2015;36(01):181-186.

[33] Zheng J, Wittouck S, Salvetti E, Franz C, Harris H, Mattarelli P, O’Toole PW, Pot B, Vandamme P, Walter J, et al. A taxonomic note on the genus Lactobacillus: description of 23 novel genera, emended description of the genus Lactobacillus Beijerinck 1901, and union of Lactobacillaceae and Leuconostocaceae. Int J Syst Evol Microbiol. 2020;70(4):70. doi:10.1099/ijsem.0.004107. 32293557

[34] Yang D, Yu X, Wu Y, Chen X, Wei H, Shah NP, Xu F. Enhancing flora balance in the gastrointestinal tract of mice by lactic acid bacteria from Chinese sourdough and enzyme activities indicative of metabolism of protein, fat, and carbohydrate by the flora. J Dairy Sci. 2016;99(10):7809–7820. doi:10.3168/jds.2016-11467. 27448855

[35] Oh D-R, Kim JR, Choi CY, Choi C-H, Na C-S, Kang BY, Kim S-J, Kim YR. Effects of ChondroT on potassium Oxonate-induced Hyperuricemic mice: downregulation of xanthine oxidase and urate transporter 1. BMC Complement Altern Med. 2019;19(1):10. doi:10.1186/s12906-018-2415-2. 30621705 PMC6323677

[36] Wang W, Zhou Q, Dai W, Qiu C, Wang H, Xinguo LU, Lin S, Jinghua Y, Zhuoya M. Alterations of gut microbiota in infants with refractory epilepsy. Chinese Journal of Microecology. 2017;29(05):502-505.

[37] Luk AJ, Simkin PA. Epidemiology of hyperuricemia and gout. Am J Manag Care. 2005;11:S435–S42. 16300457

[38] Wortmann RL. Gout and hyperuricemia. Curr Opin Rheumatol. 2002;14(3):281–286. doi:10.1097/00002281-200205000-00015. 11981327

[39] Zhi XY, Li CT, Hozzein WN, Chu X, Hu QW. Arthrobacter deserti sp. Nov., isolated from a desert soil sample. Int J Syst Evol Microbiol. 2016;111(12):2303-2310. 10.1099/ijsem.0.000986 26908080

[40] Chen Y, Wu H, Lo H, Lin W, Hsu W, Lin C, Lin P, Yanagida F. Isolation and characterisation of lactic acid bacteria from jiang-gua (fermented cucumbers), a traditional fermented food in Taiwan. J Sci Food Agric. 2012;92(10):2069-2075. 10.1002/jsfa.5583 22271629

[41] Tsuboi H, Kaneko N, Satou A, Tsuchiya Y LACTIC ACID BACTERIA HAVING ACTION OF LOWERING BLOOD URIC ACID LEVEL. 2012.

[42] Kim HJ, Lee JS, Chung HY, Song SH, Suh H, Noh JS, Song YO. 3-(4′-Hydroxyl-3′,5′-dimethoxyphenyl)propionic Acid, an Active Principle of Kimchi, Inhibits Development of Atherosclerosis in Rabbits. J Agric Food Chem. 2007;55(25):10486–10492. doi:10.1021/jf072454m. 18004805

[43] Tapsell LC. Fermented dairy food and CVD risk. British Journal of Nutrition. 2015;113(Suppl S2):S131. doi:10.1017/S0007114514002359. 26148916

[44] Liu M, Lu X, Khan A, Ling Z, Wang P, Tang Y, Liu P, Li X. Reducing methylmercury accumulation in fish using Escherichia coli with surface-displayed methylmercury-binding peptides. J Hazard Mater. 2019;367:35–42. doi:10.1016/j.jhazmat.2018.12.058. 30594015

[45] 马卓, 龚书识, 苏林冲, 向诗非, 袁林, 代玉芳, et al. 痛风现状及其患者依从性情况. 世界最新医学信息文摘 2018.

[46] Li X, Li J, Li M, Meng X. [Screening and functional properties of cholesterol-degrading lactic acid bacteria from Jiangshui]. Wei Sheng Wu Xue Bao = Acta Microbiologica Sinica. 2015;55:1001–1009. 26665597

[47] Potrikus C, Breznak JA. Anaerobic Degradation of Uric Acid by Gut Bacteria of Termites †. Appl Environ Microbiol. 1980;40(1):125–132. doi:10.1128/AEM.40.1.125-132.1980. 16345588 PMC291535

[48] Martinon F. Mechanisms of uric acid crystal-mediated autoinflammation. Immunol Rev. 2010;233:218–232. 20193002 10.1111/j.0105-2896.2009.00860.x

[49] Iwadate Y, Kato JI. Identification of a Formate-Dependent Uric Acid Degradation Pathway in Escherichia coli. J Bacteriol. 2019;11:201. 10.1128/JB.00573-18 PMC6509651 30885932

[50] Schultz AC, Nygaard P, Saxild HH. Functional Analysis of 14 Genes That Constitute the Purine Catabolic Pathway in Bacillus subtilis and Evidence for a Novel Regulon Controlled by the PucR Transcription Activator. J Bacteriol. 2001;183:3293–3302. 11344136 10.1128/JB.183.11.3293-3302.2001 PMC99626

[51] Ramazzina I, Folli C, Secchi A, Berni R, Percudani R. Completing the uric acid degradation pathway through phylogenetic comparison of whole genomes. Nat Chem Biol. 2006;2:144–148. 16462750 10.1038/nchembio768

[52] Martins FS, Elian SD, Vieira AT, Tiago FC, Martins AK, Silva FC, Souza ÉL, Sousa LP,Araújo HR, Pimenta PF. Oral treatment with Saccharomyces cerevisiae strain UFMG 905 modulates immune responses and interferes with signal pathways involved in the activation of inflammation in a murine model of typhoid fever. International Journal of Medical Microbiology. 2011;301:359–364. 21236729 10.1016/j.ijmm.2010.11.002

[53] Chen RJ, Chen MH, Chen Y, Hsiao CM, Chen HM, Chen SJ, Der WM, Yech, YJ, Yuan G, Wang Y. Evaluating the urate-lowering effects of different microbial fermented extracts in hyperuricemic models accompanied with a safety study. Journal of Food and Drug Analysis. 2017;25:597–606. 28911646 10.1016/j.jfda.2016.07.003 PMC9328828

[54] Balbi A, Ponce D, Abrão J. Early initiation of dialysis: mortality and renal function recovery in acute kidney injury patients. Jornal Brasileiro De Nefrologia:’orgao Oficial De Sociedades Brasileira E Latino-Americana De Nefrologia. 2012;34:337–342. 23318821 10.5935/0101-2800.20120022

[55] Lu H, Ning X, Chen Y, Han S, Chi P, Zhu S, Yue Y. Predictive value of serum creatinine, blood urea nitrogen, uric acid, and β2-microglobulin in the evaluation of acute kidney injury after orthotopic liver transplantation. Chin Med J. 2018;131:1059. 29692377 10.4103/0366-6999.230726 PMC5937314

[56] Wang M, Zhao J, Zhang N, Chen J. Astilbin improves potassium oxonate-induced hyperuricemia and kidney injury through regulating oxidative stress and inflammation response in mice. Biomedicine & Pharmacotherapy. 2016;83:975–988. 27522260 10.1016/j.biopha.2016.07.025

[57] Mehmood A, Zhao L, Wang C, Nadeem M, Raza A, Ali N, Shah AA. Management of hyperuricemia through dietary polyphenols as a natural medicament: a comprehensive review. Crit Rev Food Sci Nutr. 2019;59:1433–1455. 29278921 10.1080/10408398.2017.1412939

[58] García-Arroyo FE, Gonzaga G, Muñoz-Jiménez I, Blas-Marron MG, Silverio O, Tapia E, Soto V, Ranganathan N, Ranganathan P, Vyas U. Probiotic supplements prevented oxonic acid-induced hyperuricemia and renal damage. PloS One. 2018;13:8. 10.1371/journal.pone.0202901 PMC6108486 30142173

[59] Mumford SL, Dasharathy SS, Pollack AZ, Perkins NJ, Schisterman EF. Serum uric acid in relation to endogenous reproductive hormones during the menstrual cycle: findings from the BioCycle study. Human Reproduction. 2013;28:(7):1853-1862. 10.1093/humrep/det085 PMC3685334 23562957

[60] Vieira AT, Galvo I, Macia LM, Sernaglia éM, Vinolo MAR, Garcia CC, Tavares LP, Amaral FA, Lirlndia PS,Martins FS. Dietary fiber and the short‐chain fatty acid acetate promote resolution of neutrophilic inflammation in a model of gout in mice. J Leukoc Biol. 2017;101(1):101. 10.1189/jlb.3A1015-453RRR 27496979

[61] Ríos-Covián D, Ruas-Madiedo P, Margolles A, Gueimonde M. de los Reyes-Gavilán CG, Salazar N. Intestinal Short Chain Fatty Acids and Their Link with Diet and Human Health. Frontiers in Microbiology. 2016;7:185. 26925050 10.3389/fmicb.2016.00185 PMC4756104

[62] Lozupone CA, Stombaugh JI, Gordon JI, Jansson JK, Knight R. Diversity, stability and resilience of the human gut microbiota. Nature. 2012;489:220–230. 22972295 10.1038/nature11550 PMC3577372

[63] Mikayla FA, Baxter, Ruben, Merino G, Juan D, Latorre,Brittany D, Mahaffey, Yichao, Yang. Optimizing Fluorescein Isothiocyanate Dextran Measurement As a Biomarker in a 24-h Feed Restriction Model to Induce Gut Permeability in Broiler Chickens. Frontiers in Veterinary Science. 2017;4:56. 10.3389/fvets.2017.00056 PMC5396023 28470003

[64] Russel LI, Yang L, Gaseene S, Rebecca A, Doan TH, Ross B, Lui EY,Morrow CA, Fraser JA, Kirsten N. Characterization of the Complete Uric Acid Degradation Pathway in the Fungal Pathogen Cryptococcus neoformans. Plos One. 2013;8:e64292. 23667704 10.1371/journal.pone.0064292 PMC3646786

[65] Abdolmaleki A, Maafi ZT, Dastjerdi HR, Naseri B, Ghasemi A. Immune defense of Pieris brassicae larvae in challenged with Heterorhabditis bacteriophora, its symbiotic bacteria and metabolites. Invertebrate Survival Journal. 2017;14:73–84.

[66] Guo Z, Zhang J, Wang Z, Ang KY, Huang S, Hou Q, Su X,Qiao J, Zheng Y, Wang L. Intestinal microbiota distinguish gout patients from healthy humans. Sci Rep. 2016;6:20602. 26852926 10.1038/srep20602 PMC4757479

[67] Ley RE, Turnbaugh PJ, Klein S, Gordon JI. Microbial ecology: human gut microbes associated with obesity. Nature. 2006;444:1022–1023. 17183309 10.1038/4441022a

[68] Tian X, Yu Z, Feng P, Ye Z, Li R, Liu J, Hu J, Kakade A, Liu P, Li X. Lactobacillus plantarum TW1-1 Alleviates Diethylhexylphthalate-Induced Testicular Damage in Mice by Modulating Gut Microbiota and Decreasing Inflammation. Front Cell Infect Microbiol. 2019;9:221. 31297340 10.3389/fcimb.2019.00221 PMC6607032

[69] Herp S, Brugiroux S, Garzetti D, Ring D, Jochum LM, Beutler M, Eberl C, Hussain S, Walter S, Gerlach RG. Mucispirillum schaedleri Antagonizes Salmonella Virulence to Protect Mice against Colitis. Cell Host Microbe. 2019;25(5):681-694.e8. 10.1016/j.chom.2019.03.004 31006637

[70] Liu J, Yu C, Li R, Liu K, Jin G, Ge R,Tang F, Cui S. High-altitude Tibetan fermented milk ameliorated cognitive dysfunction by modified gut microbiota in Alzheimer’s disease transgenic mice. Food Funct. 2020;11(6). 10.1039/c9fo03007g 32458851

[71] Yamada N, Iwamoto C, Nakamura M, Soeda M, Tsuboi H, Kano H, et al. Reducing effect of Lactobacillus gasseri PA-3 on the absorption of food-derived purines. 2016.

[72] Leverett J, Mayne J, Missler S, Scimeca J, Roth R. Methods for scavenging oxidizing nitrogen and oxygen species with fragrances having antioxidative properties. CN. 2006.

[73] Mahbub M, Yamaguchi N, Takahashi H, Hase R, Yamamoto H, Kikuchi S, Tanabe T. Relationship of reduced glomerular filtration rate with alterations in plasma free amino acids and uric acid evaluated in healthy control and hypertensive subjects. Sci Rep. 2019;9:1–9. 31311955 10.1038/s41598-019-46598-7 PMC6635408

[74] Maiuolo J, Oppedisano F, Gratteri S, Muscoli C, Mollace V. Regulation of uric acid metabolism and excretion. Int J Cardiol. 2016;213:8–14. 26316329 10.1016/j.ijcard.2015.08.109

[75] Wooden J, Kyes S, Sibley CH. PCR and strain identification in Plasmodium falciparum. Parasitology Today. 1993. 10.1016/0169-4758(93)90131-x 15463789

[76] Lin D, Zhu S, Chen Y, Huang Y, Yang J, Chen J. Paracoccus indicus sp. nov., isolated from surface seawater in the Indian Ocean. Antonie Van Leeuwenhoek. 2019;112(6):927-933. 10.1007/s10482-019-01226-2 30737708

[77] Thompson J. improving the sensitivity of progressive multiple sequence alignment through sequence weighting, position-specific gap penalties and weight matrix choice. Nucleic Acids Res. 1994;22(22):4673-80. 10.1093/nar/22.22.4673 PMC308517 7984417

[78] Koichiro T, Glen S, Daniel P, et al. MEGA6: molecular Evolutionary Genetics Analysis Version 6.0. Mol Biol Evol. 2013;30(12):2725-9. 10.1093/molbev/mst197 PMC3840312 24132122

[79] Li L, Teng M, Liu Y, Qu Y, Zhang Y, Lin F, Wang D. Anti-gouty arthritis and antihyperuricemia effects of sunflower (Helianthus annuus) head extract in gouty and hyperuricemia animal models. Biomed Res Int. 2017;2017. 10.1155/2017/5852076 PMC5591994 28929115

[80] Matsuda K, Tsuji H, Asahara T, Matsumoto K, Takada T, Nomoto K. Establishment of an analytical system for the human fecal microbiota, based on reverse transcription-quantitative PCR targeting of multicopy rRNA molecules. Appl Environ Microbiol. 2009;75:1961–1969. 19201979 10.1128/AEM.01843-08 PMC2663197

[81] Castillo M, Martín-Orúe SM, Manzanilla EG, Badiola I, Martín M, Gasa J. Quantification of total bacteria, enterobacteria and lactobacilli populations in pig digesta by real-time PCR. Vet Microbiol. 2006;114:165–170. 16384658 10.1016/j.vetmic.2005.11.055

[82] Ornellas RM, Santos TT, Arcucio LB, Sandes SH, Oliveira MM, Dias CV, de Carvalho SS, Uetanabaro AP, Vinderola G, Nicoli JR. Selection of lactic acid bacteria with probiotic potential isolated from the fermentation process of “Cupuaçu”(Theobroma grandiflorum); Advances in Microbiology, Infectious Diseases and Public Health: Springer:2017;973:1-16. 10.1007/5584_2017_5 28224483

[83] Mc A. a SMM-O, A EGM, B IB, a MM, A JG. Quantification of Total Bacteria, Enterobacteria and Lactobacilli Populations in Pig Digesta by Real-time PCR - ScienceDirect. Veterinary Microbiology. 2006;114:165–170. 10.1016/j.vetmic.2005.11.055 16384658

[84] Haenen D, Zhang J, Souza SC, Bosch G, Van MI, Van AJ, van BJ, Perez GO, Smidt H, Kemp B. A Diet High in Resistant Starch Modulates Microbiota Composition, SCFA Concentrations, and Gene Expression in Pig Intestine. J Nutri. 2013;143:274–283. 10.3945/jn.112.169672 23325922

[85] Liu W, Zhu W, Yao W, Mao S. Isolation and identification of a lactate-utilizing, butyrate-producing bacterium and its primary metabolic characteristics. Wei Sheng Wu Xue Bao= Acta Microbiologica Sinica. 2007;47:435–440. 17672301

[86] Duncan SH, Louis P, Thomson JM, Flint HJ. The role of pH in determining the species composition of the human colonic microbiota. Environ Microbiol. 2010;11(8):2112-22. 10.1111/j.1462-2920.2009.01931.x 19397676

[87] Caporaso JG, Lauber CL, Walters WA, Berg-Lyons D. Huntley J. Fierer N, Owens SM, Betley J, Fraser L, Bauer M, et al. Ultra-high-throughput microbial community analysis on the Illumina HiSeq and MiSeq platforms. Isme Journal. 2012;6(8):1621-4.. 10.1038/ismej.2012.8 PMC3400413 22402401

[88] .Li X, Rui J, Mao Y, Anthony Y, Roderick M. Dynamics of the bacterial community structure in the rhizosphere of a maize cultivar. Soil Biol Biochem. 2014;68:392–401.

[89] Li H, Li T, Huan L, Beasley DE, Petr H, Xiao Z, Zhang S, Li J, Lin Q, Li X. Diet Diversity Is Associated with Beta but not Alpha Diversity of Pika Gut Microbiota. Front Microbiol. 2016;7:1169. 10.3389/fmicb.2016.01169 PMC4961685 27512391

[90] Mago T, Salzberg SL. FLASH: fast Length Adjustment of Short Reads to Improve Genome Assemblies. Bioinformatics. 2011;27:2957–2963. 21903629 10.1093/bioinformatics/btr507 PMC3198573

[91] Leray M, Meyer CP, Mills SC. Metabarcoding dietary analysis of coral dwelling predatory fish demonstrates the minor contribution of coral mutualists to their highly partitioned, generalist diet. PeerJ. 2015;3:e1047. 26137428 10.7717/peerj.1047 PMC4485734

[92] Callahan BJ, McMurdie PJ, Rosen MJ, Han AW, Johnson AJ, Holmes SP. DADA2: high-resolution sample inference from Illumina amplicon data. Nat Methods. 2016;13(7):581-3. 10.1038/nmeth.3869 PMC4927377 27214047

