# Supplementary material for: Limosilactobacillus fermentum JL-3 isolated from “Jiangshui” ameliorates hyperuricemia by degrading uric acid
Source: Gut Microbes. 2021 Mar 25;13(1):1897211. doi: 10.1080/19490976.2021.1897211 (PMC8007157; doi:10.1080/19490976.2021.1897211)
Supplement: Supplemental Material [file KGMI_A_1897211_SM3689.zip › Supplement File 1.docx]

Investigation on incidence rate of food paste water and gout in Gansu area

We are the school of life sciences, Lanzhou University. We recently found that people in northern areas should not be affected by ventilation. We hypothesized that it might be related to the eating habits of the area, including its unique local food- “jiangshui” noodle. In order to verify our ideas, we designed this questionnaire.

We hope you can fill in this questionnaire carefully.

1. Your gender:

Male

Female

1. Your age group:

Under 18

18~25

26~30

31~40

41~50

51~60

Over 60

1. The city where you live for a long time
2. What's your weight (kg / kg)?

Less than 45

In the middle is a kilogram and a gradient

Over 100

1. What is your height (cm / cm)?

Less than 150

In the middle is a centimeter and a gradient

More than 200

1. Do you like starched water? How many times a week do you eat on average in summer?

The gradient goes from zero to 20

1. Do you like starched water? How many times a week do you eat on average in summer?

The gradient goes from zero to 20

1. How often do you eat seafood a week?

The gradient goes from zero to 20

1. How many meals of meat do you eat a week on average?

The gradient goes from zero to 20

1. How often do you drink?

Don't drink

Drinking occasionally

Regular drinking

1. Do you know that higher uric acid levels will lead to gout?

Know it

Never heard of it

1. Do you have gout?

Yes, I have

No, I don't

1. According to the relatives and friends around you, do you feel that people suffering from gout who often eat jiangshui noodle?

I don't know. Nobody around me has gout

Yes, those gout patients often eat syrup water

No, those gout patients don't eat the water

High levels of uric acid can lead to gout, but many people are not in the habit of checking their uric acid, they miss the opportunity to prevent uric acid in advance. Lanzhou university can provide free hyperuricemia test.

1. Are you interested in checking your own uric acid level?

Yes

No

If you are willing to help us understand this problem, we hope you can help us complete the experiment in question. We will check your uric acid level regularly.

1. Would you like to eat water for a period of time and then check your uric acid and uric acid levels?

Yes, I would like to take part in the examination.

No
