# Supplementary material for: Limosilactobacillus fermentum JL-3 isolated from “Jiangshui” ameliorates hyperuricemia by degrading uric acid
Source: Gut Microbes. 2021 Mar 25;13(1):1897211. doi: 10.1080/19490976.2021.1897211 (PMC8007157; doi:10.1080/19490976.2021.1897211)
Supplement: Supplemental Material [file KGMI_A_1897211_SM3689.zip › Supplement File 2.docx]

Supplementary Information

Supplementary Figures

Supplementary Figure 1. The survey reports. A] The gout incidence in Gansu province. The abscissa represents the weekly consumption frequency of Jiangshui, and the ordinate represents proportion of gout patients with different consumption frequency of Jiangshui. B] Pearson correlation of the consumption of “Jiangshui” noodle and the incidence of gout. P≤0.01. Based on the proportion of the two in the population.


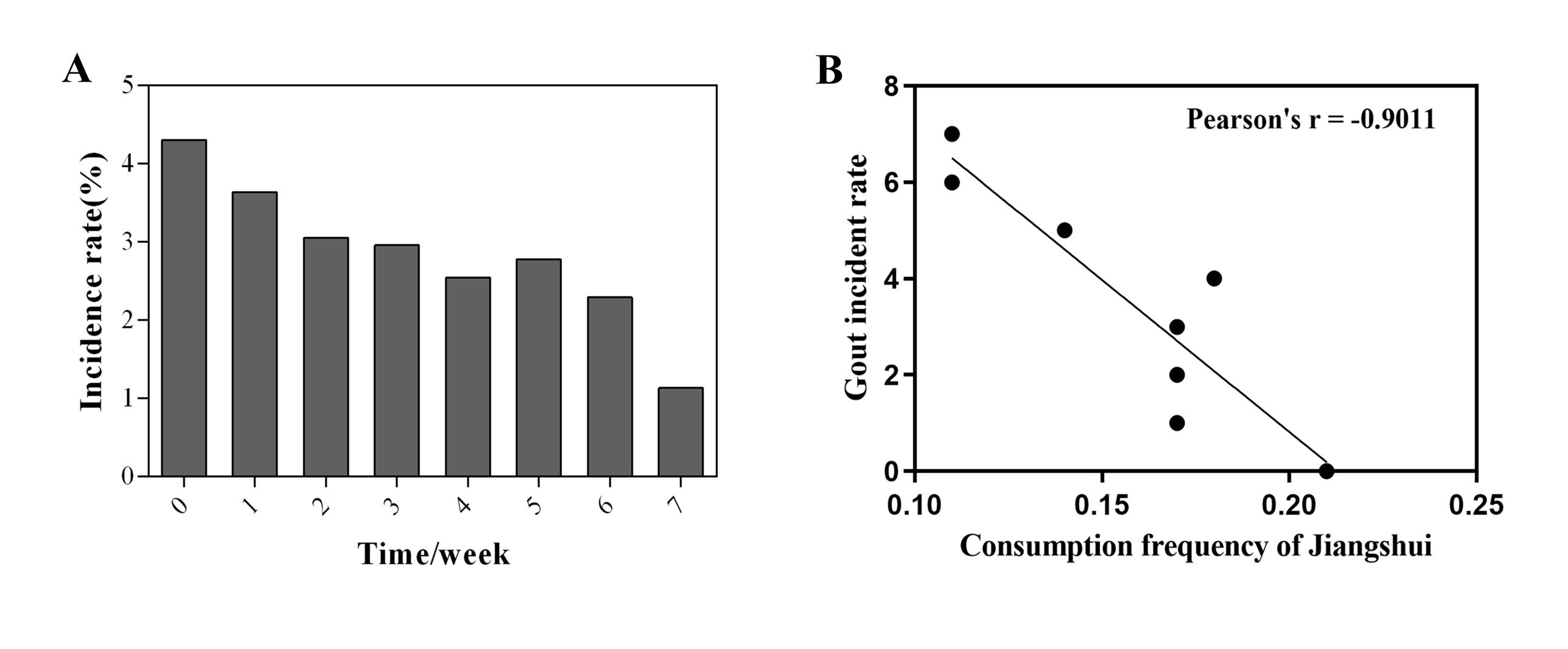


Supplementary Figure 2.A] The concentration of UA in faeces at 2, 4, 6, 8, 10, 12 days. B] The concentration of UA in urine in the whole experiment. Values are mean± SEM (n=5 per group). Bars show the mean ± SD (n=5 mice per group) **=P ﹤0.01; **** =P ﹤0.0001.


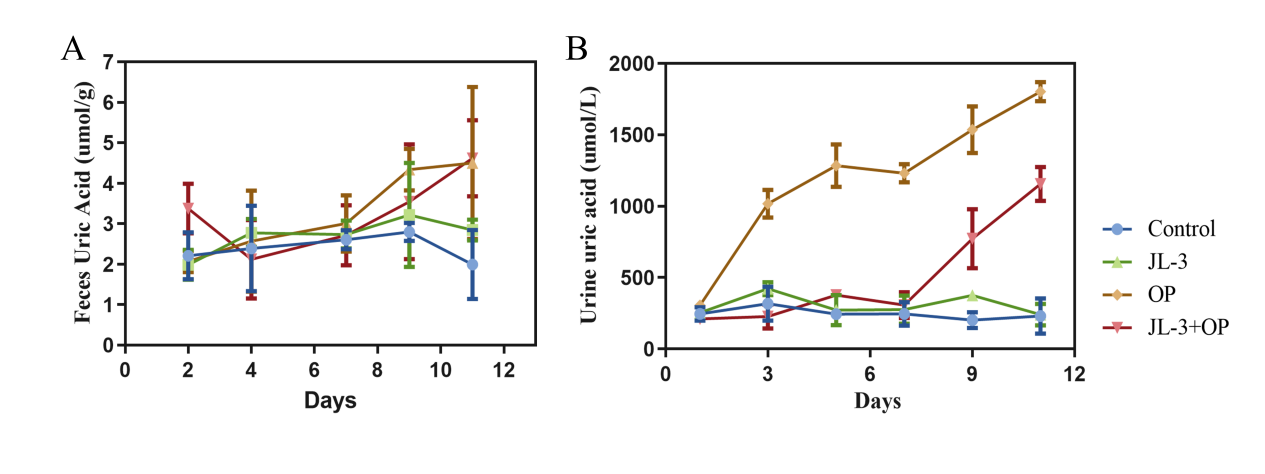


Supplementary Figure 3. Levels of IL-1β (A)、MDA (B) in mice serum in different group. C] Levels of IL-1β in mice liver. D] Content of MDA in mice liver. E] Levels of IL-1β in mice kidneys. F] Production of XOD in mice kidneys. G] Levels of MDA in mice intestinal in different group. Bars show the mean ± SD (n=5 mice per group) ** =P ﹤0.01; **** =P ﹤0.0001.





Supplementary Figure 4. Levels of UA (A)、BUN (B) and CRE (C) in mice serum in different group. D] The concentration of short-chain fatty acids in mouse feces. Bars show the mean ± SD (n=5 mice per group) **=P ﹤0.01; **** =P ﹤0.0001.


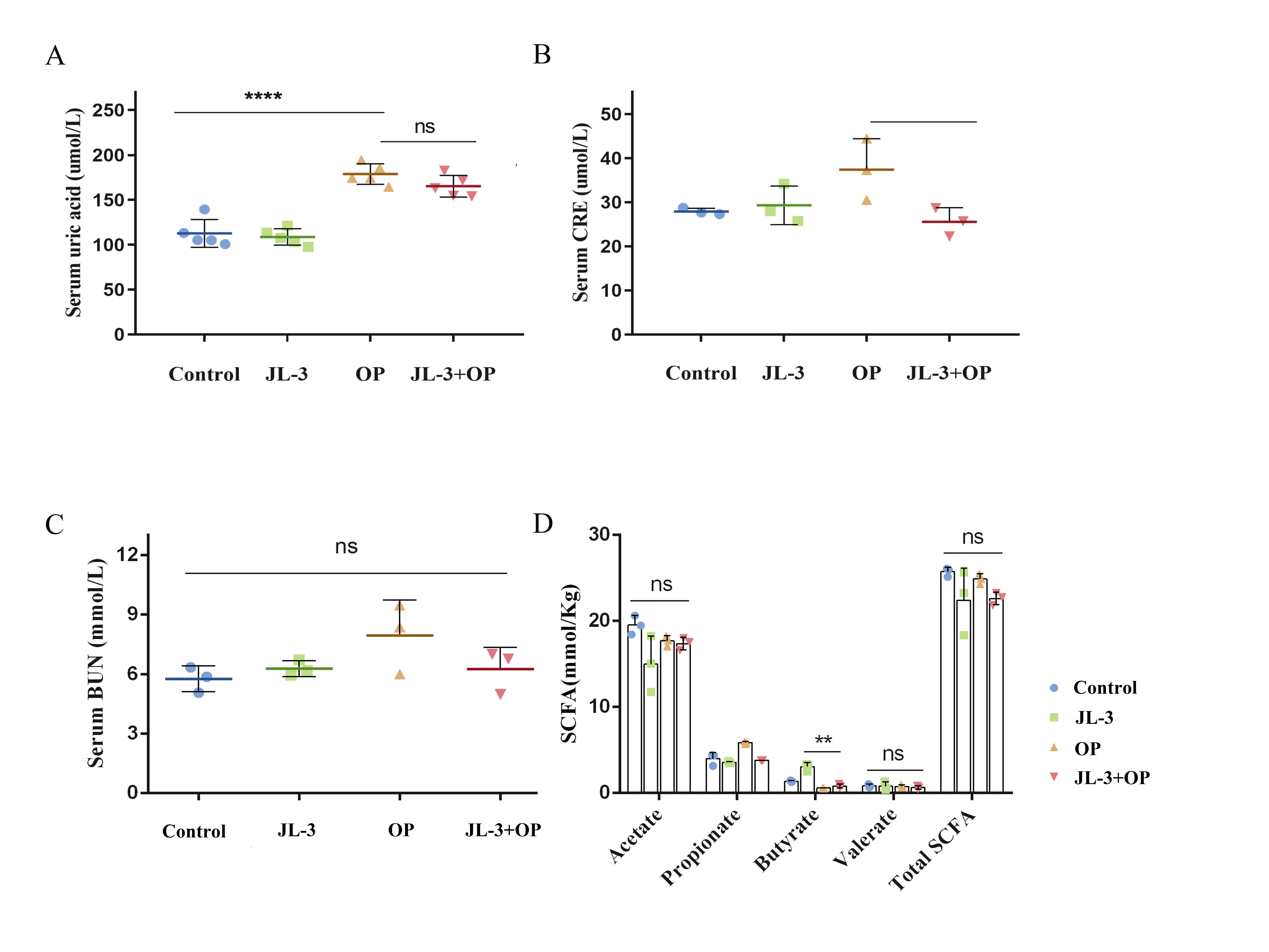


Supplementary Figure 5. JL-3 treatment restored specific microbiota changes in OP-exposed mice. A] Rarefaction Curve. B] Principle coordinates analysis (PCoA) of overall diversity based on the Bray-Curtis distances. Scatter plot of PCoA scores depicting variances derived from bacterial communities in the four groups. C] Comparison of phylum relative abundance in different groups. D] The relative abundance of the most abundant bacteria genera in the four groups. Bars show the mean ± SD (n=5 mice per group) **=P ﹤0.01; **** =P ﹤0.0001.


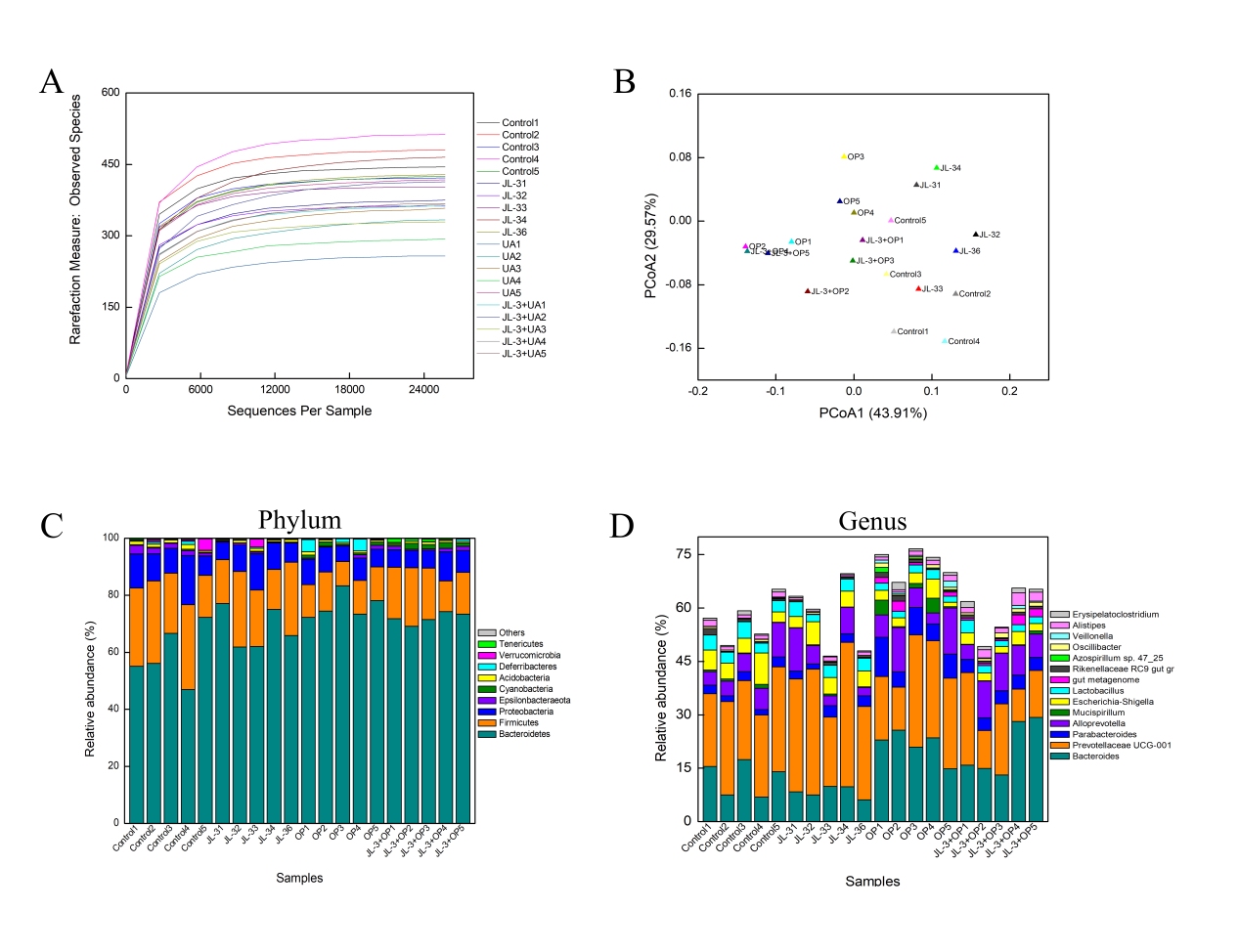


Supplementary Table 1. Alpha diversity index in six group.

| Sample-id | Shannon | Faith-pd | Observed_otus | Evenness |
| --- | --- | --- | --- | --- |
| C_1_ | 6.5951 | 30.52 | 446 | 0.749 |
| C_2_ | 6.7356 | 38.11 | 481 | 0.756 |
| C_3_ | 6.5457 | 33.37 | 420 | 0.751 |
| C_4_ | 6.4401 | 32.27 | 512 | 0.716 |
| C_5_ | 5.8867 | 37.16 | 426 | 0.674 |
| JL-3_1_ | 5.5583 | 29.73 | 375 | 0.650 |
| JL-3_2_ | 5.4013 | 30.18 | 362 | 0.635 |
| JL-3_3_ | 6.8560 | 27.01 | 403 | 0.792 |
| JL-3_4_ | 5.4270 | 36.64 | 466 | 0.612 |
| JL-3_5_ | 5.5583 | 29.73 | 375 | 0.650 |
| OP_1_ | 5.8044 | 39.26 | 397 | 0.672 |
| OP_2_ | 5.9767 | 22.27 | 311 | 0.722 |
| OP_3_ | 5.4055 | 30.31 | 353 | 0.639 |
| OP_4_ | 5.5630 | 30.78 | 355 | 0.657 |
| OP_5_ | 5.6918 | 28.38 | 341 | 0.676 |
| JL-3+OP_1_ | 6.0139 | 21.70 | 305 | 0.729 |
| JL-3+OP_2_ | 6.6806 | 27.04 | 345 | 0.792 |
| JL-3+OP_3_ | 6.3176 | 32.53 | 438 | 0.720 |
| JL-3+OP_4_ | 6.2540 | 37.77 | 369 | 0.733 |
| JL-3+OP_5_ | 6.1944 | 20.81 | 285 | 0.760 |
| UA_1_ | 4.2521 | 26.68 | 257 | 0.531 |
| UA_2_ | 4.5054 | 22.46 | 333 | 0.538 |
| UA_3_ | 5.6552 | 32.57 | 359 | 0.666 |
| UA_4_ | 5.2622 | 20.65 | 291 | 0.643 |
| UA_5_ | 6.6309 | 27.50 | 419 | 0.761 |
| JL-3+UA_1_ | 5.9133 | 23.67 | 364 | 0.695 |
| JL-3+UA _2_ | 5.3235 | 25.40 | 410 | 0.613 |
| JL-3+UA _3_ | 5.8559 | 22.51 | 331 | 0.700 |
| JL-3+UA _4_ | 4.7550 | 27.48 | 366 | 0.558 |
| JL-3+UA _5_ | 5.6540 | 23.84 | 370 | 0.663 |

Supplementary Table 2. Identification of 20 strains selected from Jiangshui by blasting in NCBI database.

| **Strain** | **Species identified** | **Homology (%)** |
| --- | --- | --- |
| JL-1 | *Acetobacter orientalis* | 100.00 |
| JL-2 | *Acetobacter orientalis* | 100.00 |
| JL-3 | *Limosilactobacillus fermentum* | 98.51 |
| JL-4 | *Weissella confusa* | 100.00 |
| JL-5 | *Limosilactobacillus fermentum* | 100.00 |
| JL-6 | *Lactiplantibacillus plantarum* | 100.00 |
| JL-7 | *Limosilactobacillus fermentum* | 99.66 |
| JL-8 | *Limosilactobacillus fermentum* | 97.33 |
| JL-9 | *Limosilactobacillus fermentum* | 100.00 |
| JL-10 | *Limosilactobacillus fermentum* | 99.89 |
| JL-11 | *Klebsiella pneumoniae* | 99.53 |
| JL-12 | *Limosilactobacillus fermentum* | 99.93 |
| JL-13 | *Escherichia coli* | 99.71 |
| JL-14 | *Escherichia coli* | 99.20 |
| JL-15 | *Escherichia coli* | 99.90 |
| JL-16 | *Klebsiella pneumoniae* | 99.53 |
| JL-17 | *Klebsiella pneumoniae* | 99.44 |
| JL-18 | *Escherichia sp.* | 99.07 |
| JL-19 | *Weissella confusa* | 99.11 |
| JL-20 | *Weissella confusa* | 98.68 |
